# Permanent cutaneous vesicostomy: a pragmatic approach to safely manage lower urinary tract dysfunction in pediatric patients with chronic and life-limiting conditions and neuropathic bladders

**DOI:** 10.3389/fped.2024.1409608

**Published:** 2024-06-25

**Authors:** Santiago Vallasciani, Ahmed Al Saeedi, Ibrahim A. Khalil, Reem Babiker Mohamed, Eshan Muneer, Nadra Abdelmaguid, Joao Luiz Pippi Salle

**Affiliations:** ^1^ Division of Urology, Sidra Medicine, Doha, Qatar; ^2^ Weill Cornell Medical College, Doha, Qatar; ^3^ Department of Urology, Hamad Medical Corporation, Doha, Qatar; ^4^ Department of Pediatrics, Hamad Medical Corporation, Doha, Qatar

**Keywords:** vesicostomy, lower urinary tract dysfunction (LUTD), neuropathic bladder, cerebral palsy, neurogenic bladder dysfunction, chronic life-limiting disease, neuromuscular disease

## Abstract

**Introduction:**

Lower urinary tract dysfunction (LUTD) in cerebral palsy (CP) and other neuromuscular diseases can present with chronic retention that leads to hydronephrosis, recurrent urinary tract infections (UTI), and stone formation. Whenever the conservative treatment of LUTD fails for any reason, it is considered to be complicated LUTD, in which a surgical approach is warranted. Cutaneous vesicostomy (CV) is a simple, well-tolerated, and potentially reversible procedure that protects the upper tracts. We describe our experience using CV for this complex population.

**Materials and methods:**

Children with CP and other neuromuscular diseases admitted to pediatric long-term care units for palliative care between 2015 and 2019 were included in the study. They present multi-system involvement, polypharmacy, and Gross Motor Function Classification System levels of 4 or 5. We retrospectively studied this population's indications and results of CV.

**Results:**

Of the 52 admitted patients, 18 presented LUTD with UTI (*n*:18; 100%), stones (*n*:5; 28%), progressive hydroureteronephrosis (*n*:3; 17%), or stones (*n*:2; 11%). Conservative initial management (catheterizations, prophylaxis antibiotics) was effective in half the cases. The remaining nine were defined as complicated LUTD and underwent CV. After a mean follow-up of 11.3 months, the follow-up showed improved hydronephrosis in all nine (100%) patients. Recurrent UTIs were no longer seen in eight of nine patients, although three patients required bladder irrigations; bladder stones did not recur after CV; the kidney stones needed further intervention. Revision of the CV was required in two (11%) cases at 12 and 24 months postoperatively due to stoma stenosis.

**Conclusion:**

CV is a relatively simple and effective procedure representing a pragmatic solution for managing complicated LUTD in complex long-term institutionalized pediatric palliative care patients with neuropathic bladders.

## Introduction

Children with chronic life-limiting diseases and dependent on respiratory technology are a growing subset within the category of children with special health needs (CSHN). There is a general perception that the number of these children living with chronic life-limiting medical conditions is increasing due to all recent advances in medicine and medical technology. These children form a very heterogeneous group based on their diagnoses. A recent study from the United States showed that children with chronic complex conditions encompass an increasing proportion of the inpatient population and are high-intensity users of hospital resources (1).

Children with chronic and life-limiting diseases that involve multiple systems and organs are usually admitted to long-term facilities to provide palliative care when indicated. Those patients tend to have a poorer quality of life. Lower urinary tract dysfunction (LUTD) in those patients is mainly characterized by chronic retention that leads to progressive hydronephrosis, recurrent urinary tract infection (UTI), stone formation, and chronic kidney disease, all of which are complex problems to manage in this compromised population (2–5).

The management of LUTD in these patients is complex. It ranges from conservative measures, such as clean intermittent catheterization (CIC) and continuous antibiotic prophylaxis (CAP) that are attempted initially. Still, many fail due to complications or institutional difficulties in maintaining the program (4). These cases can be defined as complicated LUTD as a surgical approach is warranted to decompress the bladder and stabilize their condition after failure using conservative means. Cutaneous vesicostomy (CV) is an available option to decompress the bladder in these circumstances (6, 7).

Herein, we describe our experience using CV for this complex population. We hypothesize that CV is a practical and efficient alternative for managing neurogenic bladder, improving symptoms of LUTD, and stabilizing the upper urinary tract not only in newborns but even in older children and adolescents.

## Materials and methods

After approval from the institutional review board (IRB no. 1592907), we performed a retrospective chart review of cerebral palsy (CP) patients with LUTD managed between 2015 and 2019.

The definitions are as follows:

- LUTD was defined as chronic retention of urine observed by dribbling and or residual urine in the bladder higher than 20% of the expected bladder capacity by age (measured by ultrasound, bladder scan, and/or voiding cystourethrogram). Other ultrasound findings, such as bladder thickness of more than 5 mm and or irregularity/trabeculation of the bladder wall and stones, were also considered identifiers of LUTD. We defined complicated LUTD as those cases of LUTD that required surgical intervention after failure using conservative treatment.
- Study population: children with CP and chronic and life-limiting diseases necessitate admission to pediatric long-term care units as a step down from high dependency units to allow for continuity of care and provide palliative care when indicated. The study population shares common characteristics like multi-system involvement, polypharmacy, Gross Motor Function Classification System (GMFCS) levels of 4 or 5, frequent and prolonged hospital admissions, and the need for optimal care coordination.
- All patients were kept in diapers, and urinary tract investigations were only performed when LUTD was suspected because of recurrent febrile UTI. Ultrasound with an assessment of the post-void residual urine, voiding cystourethrogram, and nuclear scans were performed for them. Initial management included CIC, bladder washouts, and antibiotic prophylaxis. If, despite the initial management, the patient developed further UTI, they were defined as complicated LUTD and underwent CV using the Blockson technique, modified by Krahn and Johnson by advancing more of the posterior bladder wall up to the skin, and suturing the bladder detrusor to the anterior rectus fascia and skin (6).

The primary outcome was a complete resolution of the signs of complicated LUTD. Secondary outcomes were the need for further conservative measures necessary to treat the persistence of symptoms of complicated LUTD after CV.

## Results

During the period studied, 52 patients were admitted to the long-term complex care facility. Of them, 50 were at GMFCS level 4–5. Of these 50 patients, 18 (36%) presented with signs and symptoms of LUTD with one or more of the following: UTI in 18 (36%), stones in 2 (11%), or progressive hydroureteronephrosis (SFU grade 3 or 4, ureters diameter 7 mm or more) in 3 (6%). They were then categorized as symptomatic LUTD. Of them, nine (50%) developed complicated LUTD and underwent CV.

Patient demographics and clinical features are presented in Table 1. After a mean of 11.3 months after CV (range 6–24 months), all the patients were emptying the bladder well.

**Table 1:** Age and the primary diagnosis of the patients with LUTD.

Patient	Age	Primary diagnosis	LUTD complication
1	18 months	SGM-9 gene–related disorder	Recurrent UTI, hydronephrosis
2	5 years	Myelomeningocele with hydrocephalus and cerebral palsy	Recurrent UTI, hydronephrosis
3	6 years	Spinal muscular atrophy	Recurrent UTI
4	11 years	Tracheolaryngomalacia, chronic lung disease on ventilator, spina bifida occulta, Down's syndrome	Recurrent UTI
5	7 years	Myelomeningocele with hydrocephalus and cerebral palsy	Recurrent UTI, bladder and renal stone
6	12 years	Spinal muscular atrophy	Recurrent UTI, bladder and renal stone
7	7 years	CP secondary to acute disseminated encephalomyelitis	Recurrent UTI
8	5 years	CP with quadriplegia	Recurrent UTI
9	14 years	Hypoxic ischemic encephalopathy	Recurrent UTI, hydronephrosis

Preoperative hydronephrosis was reduced in all the patients after CV. Five patients (55%) had complete resolution of recurrent UTIs without further maneuvers. Three of the remaining four patients were managed using bladder irrigation with gentamycin for 7 days followed by normal saline irrigation through vesicostomy to avoid further UTI. Bladder stones were removed at the time of vesicostomy and none recurred; however, the kidney stones were managed separately later. Revision of the vesicostomy was required in two (11%) cases at 12 and 24 months postoperatively due to stoma stenosis. No other significant complications (Clavien–Dindo grade 2 and above) were recorded.

**Figure 1:**
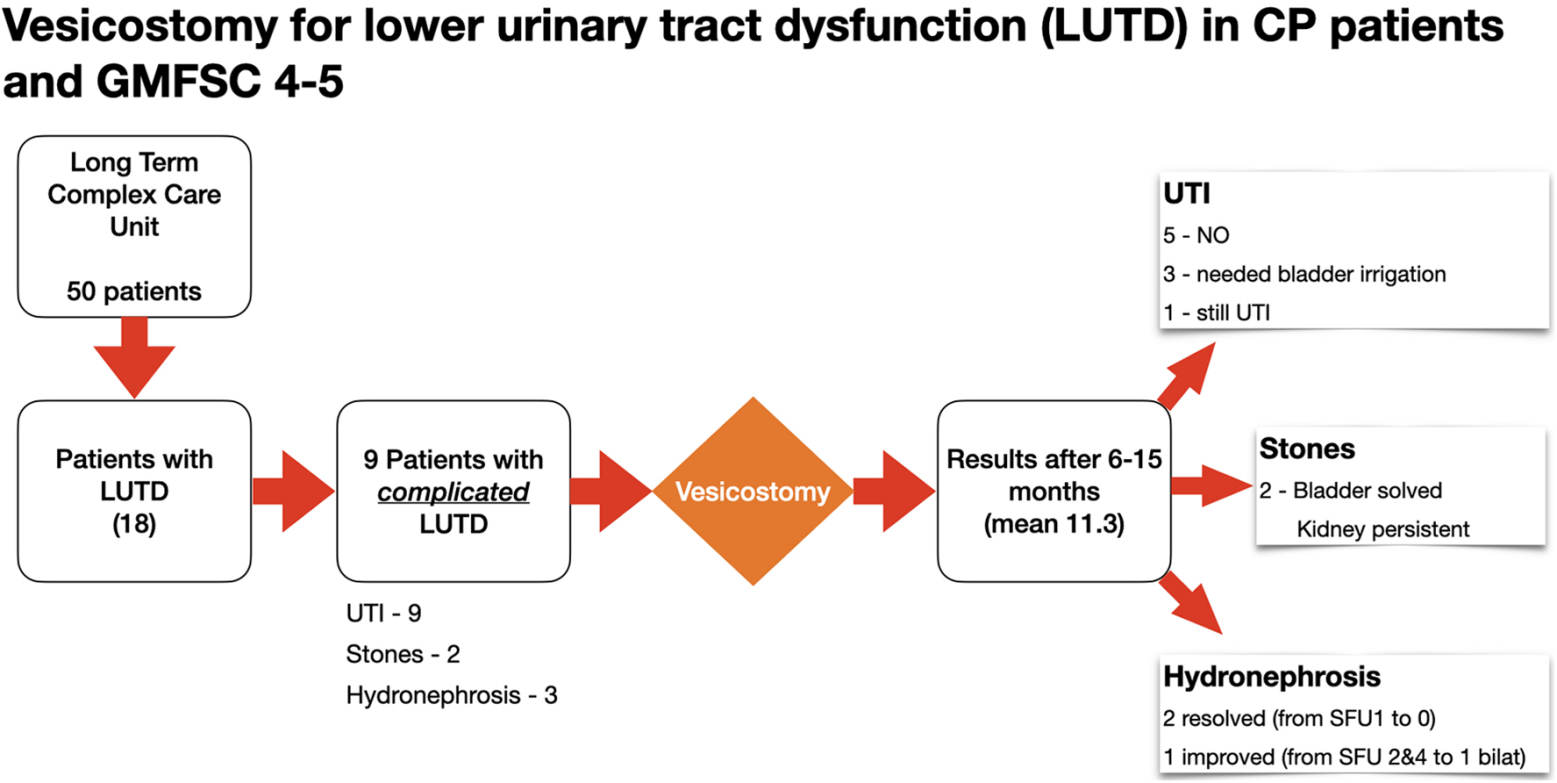
Flow chart of patients included in the study and the outcome.

The flow chart of patients included in the study is outlined in Figure 1.

## Discussion

LUTD is not uncommon among patients with CP. In their review, Samijn et al. (2) found that about half of patients with CP present LUTD secondary to detrusor overactivity rather than pelvic floor overactivity. The former has a higher impact in the upper tracts with hydronephrosis, secondary reflux, and pyelonephritis. It seems more frequent in high levels of GMFCS, as observed by Bross et al. (3). LUTD can be classified according to the cause for neurogenic and non-neurogenic LUTD (8); in our population the presence of high GMFCS levels in 50 out of 52 patients explains why symptomatic LUTD was present in 36% and half of them were complicated LUTD. In addition, as our population had neuroanatomic or neuromuscular diseases, a neuropathic etiology was proposed even if urodynamic studies were not carried out in our study population, as invasive urodynamics is difficult to perform or interpret in this fragile bedridden population.

The management of LUTD in this complicated patient category varies and ranges from review and modification of patients’ medications, such as discontinuation of anticholinergic medications or addition of alpha-adrenergic blockers, along with other conservative measures, such as CIC to CV or even more sophisticated alternatives such as the construction of abdominal catheterizable stomas (Mitrofanoff channel). However, they are more complex procedures that require significant involvement of specialized care and CIC (9, 10).

The CV, as described by Blockson and modified by Krahn and Johnson (6), is a relatively simple surgical procedure to treat LUTD in complex patients with minimal morbidity (7, 11). The modification of the CV by Krahn and Johnson results in fewer stomal complications, such as stenosis and prolapse, and reduces the need for intermittent stoma catheterization (6). The CV also presents stable results over time with reduced stoma stenosis. In our series, the two cases that needed surgical revision were both post-pubertal patients. As previously reported, it may be hypothesized that skin healing characteristics may have played a role (12). Another possible reason for stenosis/CV revision in older patients, as in our cases, is the larger size of the patient and the need to span a longer distance from the retropubic space to the skin. This could affect the vascularity of the exposed stoma.

As seen in our study, CV was instrumental in decompressing the urinary tract and significantly diminishing the number of recurrent UTIs. In addition, it allowed for irrigation of the bladder with antibiotics through the stoma in the more recalcitrant cases. Salih et al. found similar results in the resolution of UTI, hydronephrosis, and improvement of functional outcome after CV for bladder outlet obstruction in patients with CP (11).

Although the CV is safe and effective, it should not be considered for all patients with neurogenic bladders who preferably should be decompressed using CIC with or without continent catheterizable stoma (9, 10). However, in our particular population of institutionalized long-term care patients, often with other significant comorbidities, those complex procedures are risky and demand special postoperative care (1, 5).

The present study has some limitations. These include the small number of patients, the retrospective design, and that no patient underwent urodynamics studies; however, it provides a pragmatic alternative for such a complex and unfortunate population. The initial results encourage us to propose CV for management and possible validation by other healthcare providers involved in the care of long-term institutionalized patients with LUTD.

## Conclusion

CV is a simple and effective procedure for complicated LUTD in complex institutionalized long-term patients with neuropathic bladders. As well as effective urinary decompression, it also provides alternative access for bladder washouts whenever necessary for the management of recurrent UTIs.

## Data Availability

The original contributions presented in the study are included in the article/Supplementary Material, further inquiries can be directed to the corresponding author.
